# Melatonin Improves Drought Stress Tolerance by Remodeling Lipid Metabolism in *Setaria italica* L.

**DOI:** 10.3390/plants14213314

**Published:** 2025-10-30

**Authors:** Jianhong Ren, Tao Zhang, Xin Yin, Yijia Zhao, Fanyi Meng, Xiaoxiao Yang

**Affiliations:** 1 College of Life Sciences, Shanxi Agricultural University, Taigu, Jinzhong 030800, China; sxau_zt@163.com; 2 College of Agriculture, Shanxi Agricultural University, Taigu, Jinzhong 030800, China; sxau_yx@163.com (X.Y.); sxau_zyj@163.com (Y.Z.); sxau_mfy@163.com (F.M.)

**Keywords:** photosynthesis, oxidative damage, glycolipids, phospholipids, unsaturated fatty acid

## Abstract

Membrane lipid remodeling represents a crucial adaptive mechanism for plants in response to drought stress. This study investigated the regulatory influence of melatonin on the photosynthetic attributes, oxidative damage, and lipid metabolism of foxtail millet seedlings subjected to drought stress, with particular emphasis on alterations in lipid composition and fatty acid unsaturation. The findings indicated that melatonin treatment markedly enhanced the drought tolerance of foxtail millet seedlings, resulting in increases in chlorophyll content, net photosynthetic rate, and total dry weight by 51.2%, 39.8%, and 51.1%, respectively. Melatonin increased the levels of monogalactosyldiacylglycerol (MGDG), digalactosyldiacylglycerol (DGDG), sulfoquinovosyldiacylglycerol (SQDG), and phosphatidylcholine (PC), while promoting the accumulation of unsaturated fatty acid (18:3) and leading to an increase in the double bond index (DBI). Concurrently, there were significant alterations in the expression of genes associated with glycolipid and phospholipid biosynthesis, aligning with the observed changes in lipid components. These findings indicate that melatonin potentially enhances the drought tolerance of foxtail millet seedlings through the regulation of lipid metabolic reprogramming. This process involves an increase in the content of unsaturated fatty acids and an optimization of the lipid unsaturation index, which collectively contribute to the greater stability, fluidity, and integrity of cellular membranes.

## 1. Introduction

In light of the escalating challenges posed by global warming and water scarcity, water stress has emerged as a critical issue in agricultural production worldwide. This is particularly pronounced in arid and semi-arid regions, where farmers are increasingly confronted with prolonged and intensified drought events, whether seasonal or irregular [1]. Drought stress exerts numerous adverse effects on crop growth and development, including the inhibition of photosynthesis, decreased carbon assimilation efficiency, disruption of carbohydrate metabolism, accelerated protein degradation, and membrane lipid peroxidation, among other physiological and metabolic disturbances [2]. These issues collectively result in reduced biomass and yield losses. Consequently, there is an urgent imperative to enhance crop drought tolerance to address the food security challenges posed by the continuous growth of the global population. The exogenous application of plant growth regulators is considered a practical and effective strategy for improving crop drought resistance [3]. Among these regulators, melatonin, an endogenous signaling molecule ubiquitously present in plants, has demonstrated significant potential for application in recent years [4].

Melatonin was initially identified in the pineal gland of cattle in 1958 and subsequently recognized as a ubiquitous compound present across animals, plants, and microorganisms [5]. In the animal kingdom, melatonin serves as a pivotal hormone involved in the regulation of circadian rhythms, facilitation of sleep, enhancement of immune functions, and retardation of aging, among other physiological roles [6]. In the plant domain, melatonin acts as a conserved endogenous signaling molecule and growth regulator. Its chemical structure is classified within the indole group of compounds, structurally similar to the plant hormone auxin and thus involved in regulating various plant life processes [7]. Numerous studies have demonstrated that melatonin is involved in the regulation of seed germination, root development, photosynthesis, flowering, fruit ripening, and senescence [8,9]. Furthermore, melatonin is instrumental in mediating plant responses to abiotic and biotic stresses, including drought, salinity, extreme temperatures, heavy metal toxicity, and pathogen invasion [10,11]. Melatonin contributes to the enhancement of plant drought tolerance through various mechanisms, including the regulation of stomatal closure, the augmentation of antioxidant enzyme activity, the stabilization of the photosynthetic apparatus, the promotion of osmotic adjustment substance accumulation, and cross-talk with signaling molecules such as abscisic acid (ABA) and hydrogen sulfide (H_2_S), which jointly modulate the expression of drought-responsive genes [12,13]. Nonetheless, additional research is required to elucidate the role of melatonin in improving plant drought tolerance and to further understand its underlying regulatory mechanisms.

Plant cell membranes, functioning as crucial semi-permeable barriers, are instrumental in maintaining intracellular homeostasis, thereby enabling resistance to drought stress [14]. The primary structural constituents of both the plasma membrane and the inner membrane are lipids, which exhibit considerable diversity, encompassing phospholipids, glycerides, glycolipids, and sphingolipids among others [15]. These lipid molecules not only constitute the fundamental framework of the membrane but also play pivotal roles in various essential biological processes, including energy storage, cell surface protection, and signal transduction [16]. Several studies have demonstrated that the relative abundance of different lipid categories directly influences the physicochemical properties of plant cell membranes, such as fluidity and permeability [17]. It is important to recognize that the lipid composition of cell membranes is not static; rather, it is substantially affected by the type of plant tissue, interspecies variations, and developmental stages [18]. Notably, various abiotic stresses, such as drought stress, can markedly alter lipid composition and content. Under drought stress, plants have been observed to exhibit significant changes in lipid accumulation, conversion of lipid components, and modifications in the saturation levels of fatty acid chains [19]. These dynamic lipid remodeling processes are recognized as essential physiological mechanisms that enable plants to perceive stress signals, modulate membrane properties, and ultimately adapt to drought stress.

In the adaptive response of plants to drought stress, the dynamic regulation of membrane lipid remodeling and lipid signaling transduction has been demonstrated to be intricately linked to drought tolerance [20]. This phenomenon has been extensively validated across various species, including sorghum [21], maize [22], and Prunus [19]. Specifically, research has identified that the accumulation levels of phosphatidylcholine (PC) and phosphatidylethanolamine (PE) in sorghum, alongside the content of digalactosyldiacylglycerol (DGDG) in maize, exhibit a significant positive correlation with plant drought tolerance. Similarly, the specific alterations in triacylglycerols (TAG) and unsaturated fatty acids levels in Prunus have been identified as critical regulatory factors in conferring resistance to drought stress. The research findings collectively suggest that key genes involved in lipid metabolism are integral to the genetic enhancement of drought tolerance in crops. For example, the overexpression of the nonspecific lipid transfer protein gene in cotton has been shown to effectively modify its membrane lipid composition, thereby significantly influencing the plant’s response to drought stress [23]. In summary, the remodeling of membrane lipids, through alterations in cellular membrane lipid components such as types, contents, and degrees of saturation, constitutes a vital adaptive mechanism that enables plants to preserve membrane integrity, maintain appropriate fluidity, and ensure overall stability under drought stress conditions.

Foxtail millet (*Setaria italica* L.) is among the most extensively cultivated and highest-yielding minor coarse grain crops globally. Characterized by a short life cycle, a compact genome, and high nutritional value, it exhibits strong adaptability and functions as a C4 plant. Consequently, foxtail millet is recognized as a model organism for investigating stress physiology [24]. In agricultural contexts, drought stress has emerged as a significant limiting factor that inhibits the growth and development of foxtail millet, adversely affecting its yield formation [25]. Drought stress during the seedling stage constitutes a prevalent form of abiotic stress, typically arising from inadequate rainfall or insufficient irrigation. This phase of drought critically inhibits root development and aboveground growth, culminating in decreased leaf area and compromised photosynthetic capacity, thereby adversely impacting the establishment and survival of juvenile plants. Ultimately, these conditions result in inadequate biomass accumulation and substantial yield reductions in crops [26,27]. Therefore, it is imperative to explore effective strategies to enhance the drought resistance of foxtail millet. Presently, the majority of research concerning the role of melatonin in increasing plant drought tolerance emphasizes its capacity to enhance antioxidant defenses, facilitate the accumulation of osmotic substances, and improve photosynthetic efficiency [12,13]. Conversely, there is less focus on its involvement in the process of membrane lipid remodeling. Membrane lipid remodeling serves as a crucial regulatory mechanism enabling plants to adapt to drought stress, primarily through the adaptive modulation of lipid composition, fatty acid unsaturation, and membrane stability [19]. Here, we hypothesize that melatonin may augment the drought resistance of foxtail millet by influencing membrane lipid metabolism. Consequently, the study investigates the effects of melatonin treatment on foxtail millet plants subjected to drought conditions, focusing on alterations in lipid content, fatty acid composition, and the expression levels of related genes. The objective is to elucidate the mechanism by which melatonin enhances drought tolerance in foxtail millet, specifically from the perspective of membrane lipid remodeling.

## 2. Results

### 2.1. Melatonin Promoted Plant Growth Under Drought Stress

**Figure 1:**
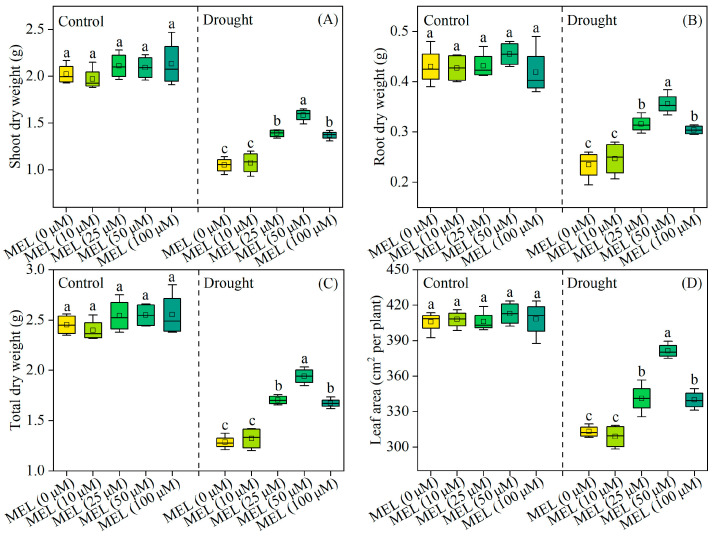
Effects of drought and melatonin on shoot dry weight (**A**), root dry weight (**B**), total dry weight (**C**), and leaf area (**D**) of foxtail millet. Values represent the mean ± SE (*n* = 4). Different letters denote significant differences among treatments (*p* < 0.05).

Under normal growth conditions, melatonin does not exhibit a growth-promoting effect on plant growth. After 12 days of drought stress treatment, there is a notable reduction in the total dry weight of foxtail millet seedlings. Conversely, the exogenous application of melatonin results in a significant increase in the total dry weight of the plants, with the most pronounced effect observed at a concentration of 50 μM/L, representing a 51.1% increase compared to treatments without melatonin (Figure 1C). Additionally, melatonin markedly enhances leaf area (Figure 1D). These findings indicate that melatonin enhances drought tolerance in foxtail millet seedlings.

### 2.2. Effects of Drought and Melatonin Treatment on Endogenous Melatonin Contents

**Figure 2:**
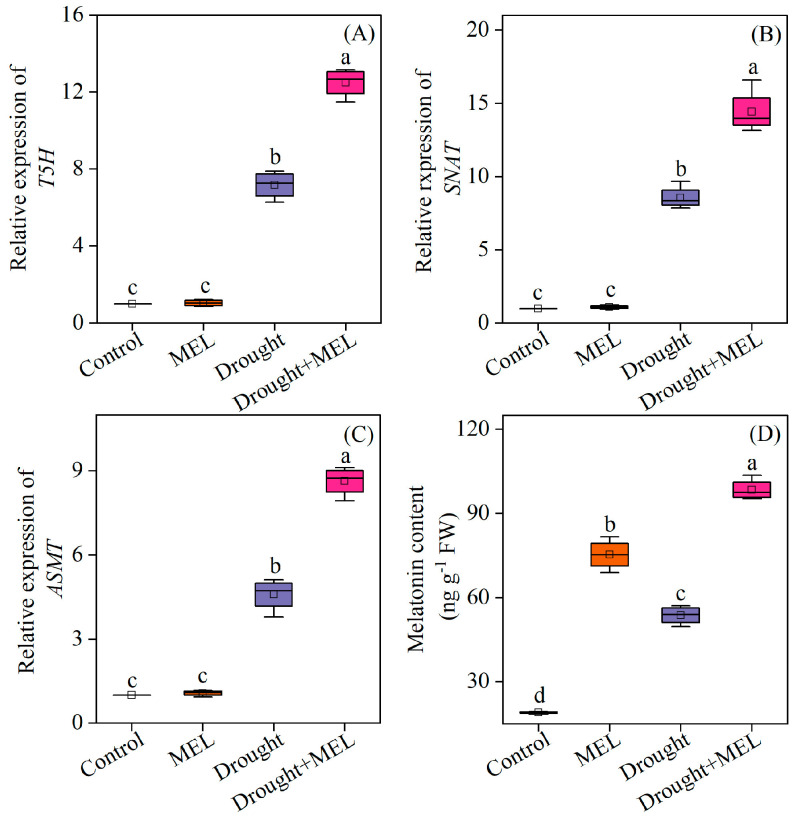
Effects of drought and melatonin on the relative expression levels of melatonin biosynthesis-related genes (**A**–**C**) and endogenous melatonin content (**D**) in leaves of foxtail millet. *T5H*: tryptamine 5-hydroxylase; *SNAT*: serotonin N-acetyltransferase; *ASMT*: Acetylserotonin O-methyltransferase. Values represent the mean ± SE (*n* = 4). Different letters denote significant differences (*p* < 0.05).

Drought stress markedly stimulated the upregulation of several critical genes involved in the melatonin biosynthesis pathway in leaves, including *tryptamine 5-hydroxylase (T5H)*, *serotonin N-acetyltransferase (SNAT)*, and *Acetylserotonin O-methyltransferase (ASMT)*. The exogenous application of melatonin further augmented the transcriptional levels of these genes (Figure 2A–C). Consequently, the endogenous melatonin content in the leaves of melatonin-treated plants exhibited a significant increase (Figure 2D). This suggests that exogenous melatonin may enhance the plant’s tolerance to drought stress by upregulating key genes in the endogenous synthesis pathway and promoting the accumulation of endogenous melatonin.

### 2.3. Melatonin Alleviated the Inhibition of Photosynthesis in Drought-Stressed Foxtail Millet Seedlings

**Figure 3:**
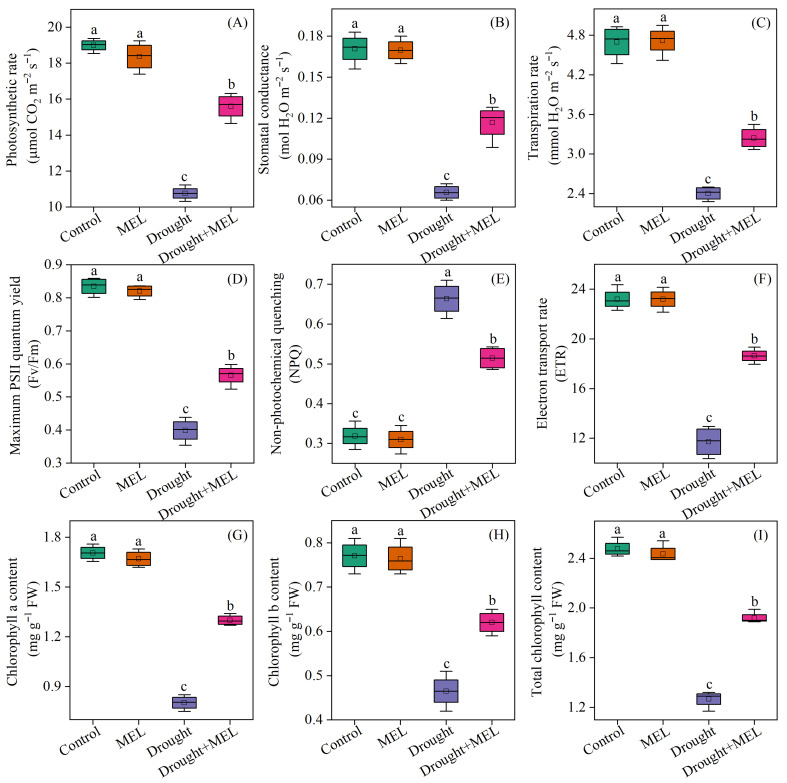
Effects of drought and melatonin on photosynthetic parameters (**A**–**C**), chlorophyll fluorescence parameters (**D**–**F**), and chlorophyll content (**G**–**I**) in leaves of foxtail millet. Values represent the mean ± SE (*n* = 4). Different letters denote significant differences (*p* < 0.05).

Under normal growth conditions, the application of exogenous melatonin did not significantly impact the photosynthetic rate, stomatal conductance, or transpiration rate of the leaves. In contrast, drought stress markedly reduced these photosynthetic parameters. Notably, melatonin application effectively mitigated the adverse effects of drought stress, as evidenced by an increase in the net photosynthetic rate, stomatal conductance, and transpiration rate by 39.8%, 71.4%, and 34.4%, respectively, after 12 days of treatment compared to the drought stress group (Figure 3A–C). Furthermore, under drought conditions, melatonin treatment significantly improved the chlorophyll fluorescence parameters and chlorophyll content of the leaves. Specifically, the Fv/Fm and chlorophyll content increased by 42.5% and 51.2%, respectively (Figure 3D,I). These findings demonstrate that melatonin mitigates drought-induced declines in photosynthetic performance by preserving the functionality of photosynthetic organs and maintaining the stability of photosynthetic pigments.

### 2.4. Melatonin Alleviated Oxidative Stress in Drought-Stressed Foxtail Millet Seedlings

**Figure 4:**
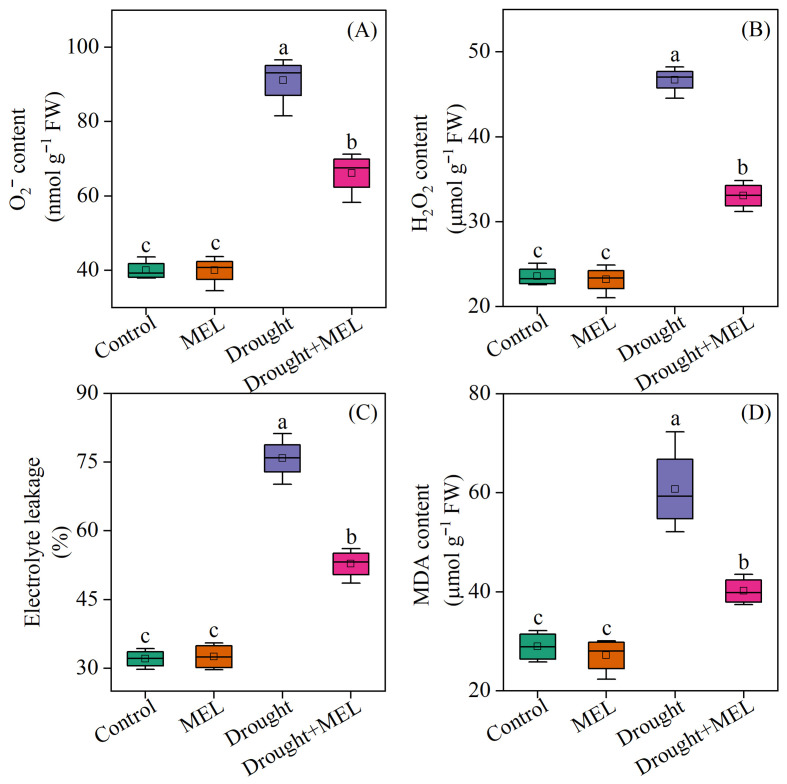
Effects of drought and melatonin on superoxide anion content (**A**), hydrogen peroxide content (**B**), electrolyte leakage (**C**), and malondialdehyde content (**D**) in leaves of foxtail millet. Values represent the mean ± SE (*n* = 4). Different letters denote significant differences (*p* < 0.05).

Under normal growth conditions, the contents of superoxide anion (O_2_^−^·), hydrogen peroxide (H_2_O_2_), malondialdehyde (MDA), and electrolyte leakage rate (EL) in plants treated with melatonin did not exhibit significant differences compared to the control group. However, exposure to drought stress markedly increased these indicators by 127.9%, 98.3%, 136.6%, and 110.1%, respectively, relative to the normal growth control group. Under drought stress conditions, the application of melatonin effectively reduced the contents of O_2_^−^·, H_2_O_2_, MDA, and EL in leaves, compared to those not treated with melatonin (Figure 4). These findings suggest that the exogenous application of melatonin alleviates oxidative damage in foxtail millet plants by reducing the accumulation of reactive oxygen species induced by drought stress.

### 2.5. Melatonin Induces Changes in Lipid and Fatty Acid Compositions in Foxtail Millet Seedlings Under Drought Stress

**Figure 5:**
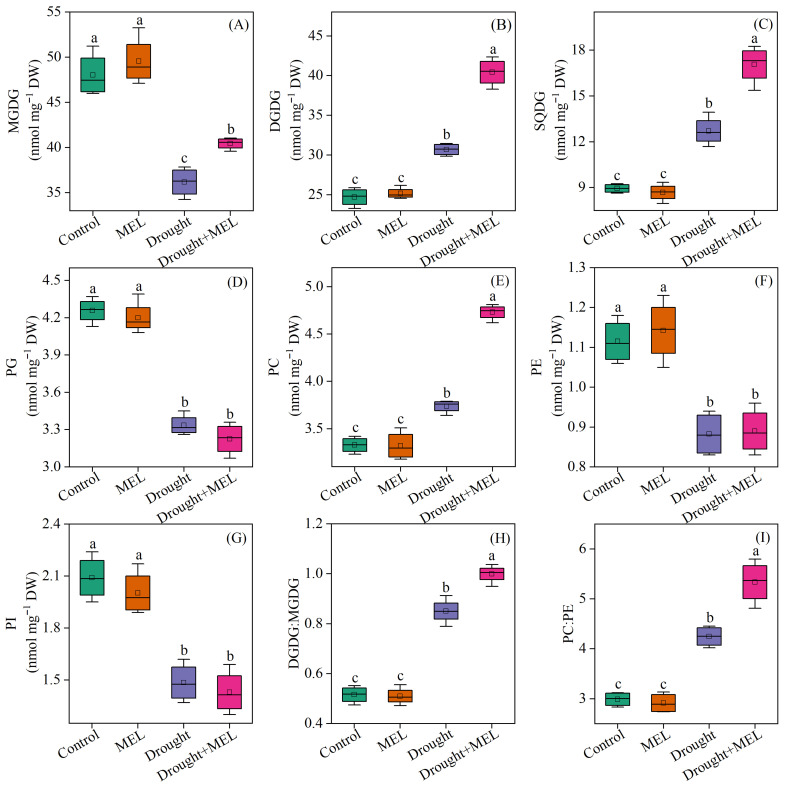
Effects of drought and melatonin on contents of MGDG (**A**), DGDG (**B**), SQDG (**C**), PG (**D**), PC (**E**), PE (**F**), PI (**G**), and ratios of DGDG:MGDG (**H**) and PC:PE (**I**) in leaves of foxtail millet. Values represent the mean ± SE (*n* = 4). Different letters denote significant differences (*p* < 0.05).

Under normal growth conditions, the application of exogenous melatonin did not exert a significant impact on the membrane lipid composition of foxtail millet leaves. Regarding glycolipid components, drought stress markedly decreased the content of monogalactosyldiacylglycerol (MGDG) while enhancing the accumulation of DGDG and sulfoquinovosyldiacylglycerol (SQDG). Following melatonin treatment, the levels of MGDG, DGDG, and SQDG all exhibited significant increases, suggesting that melatonin facilitates glycolipid biosynthesis under drought conditions (Figure 5A–C). Concerning phospholipids, drought stress resulted in a notable reduction in the levels of phosphatidylglycerol (PG), PE, and phosphatidylinositol (PI), whereas the level of PC significantly rose (Figure 5D–G). The administration of exogenous melatonin further augmented the level of PC by 26.5%, underscoring its beneficial role in sustaining phospholipid homeostasis. The drought conditions led to a notable increase in the DGDG: MGDG and PC: PE ratios. Furthermore, the exogenous application of melatonin increased these ratios even further (Figure 5H,I).

**Figure 6:**
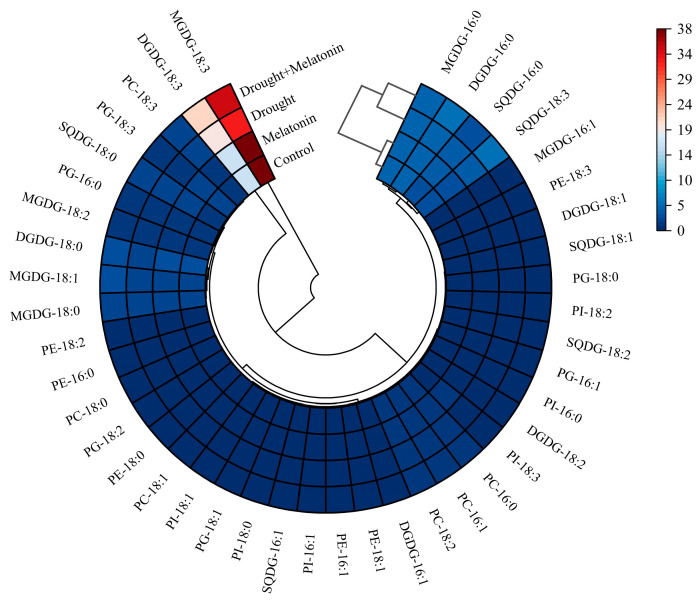
Cluster analysis of fatty acid composition in leaves of foxtail millet.

**Figure 7:**
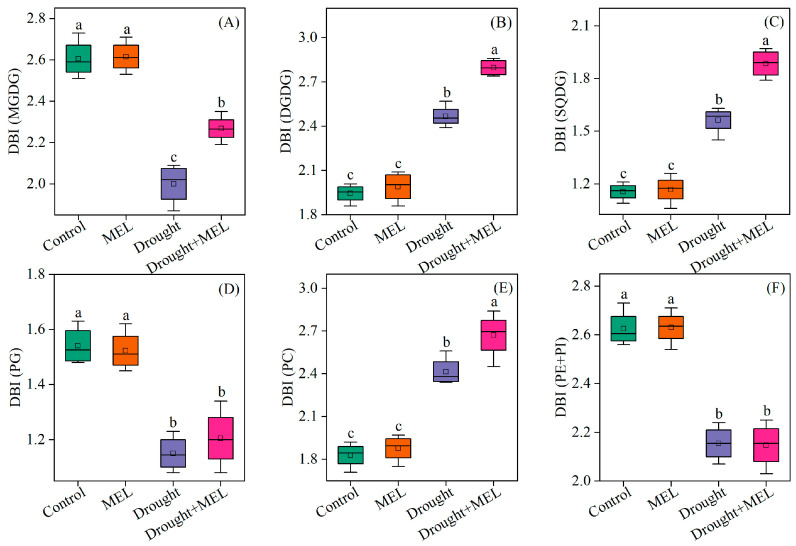
Effects of drought and melatonin on the double bond index (DBI) of MGDG (**A**), DGDG (**B**), SQDG (**C**), PG (**D**), PC (**E**), and PE + PI (**F**) in leaves of foxtail millet. Values represent the mean ± SE (*n* = 4). Different letters denote significant differences (*p* < 0.05).

From the perspective of fatty acid composition, the predominant fatty acid in MGDG and DGDG is linolenic acid (18:3). Drought stress significantly elevated the content of 18:3 in DGDG, while melatonin treatment further augmented its accumulation both in MGDG and DGDG (Table 1). Conversely, melatonin did not exert a significant effect on the content of palmitic acid (16:0) in SQDG, PG, PC, PE, and PI. However, it increased the proportion of 18:3 in SQDG and PC (Table 1). The findings from the cluster analysis further confirmed that the 18:3 in MGDG and DGDG may play a pivotal role in enhancing drought resistance as induced by melatonin (Figure 6). The DBI, a critical parameter for evaluating membrane lipid fluidity, was significantly elevated in MGDG, DGDG, SQDG, and PC under drought stress conditions. Exogenous melatonin treatment further increased the double bond index (DBI) values of these lipid components, suggesting that melatonin enhances membrane fluidity by promoting lipid unsaturation (Figure 7). This enhancement may improve plant adaptability under drought stress conditions.

### 2.6. Differentially Expressed Genes Associated with Lipid Metabolism Under Drought Stress

**Figure 8:**
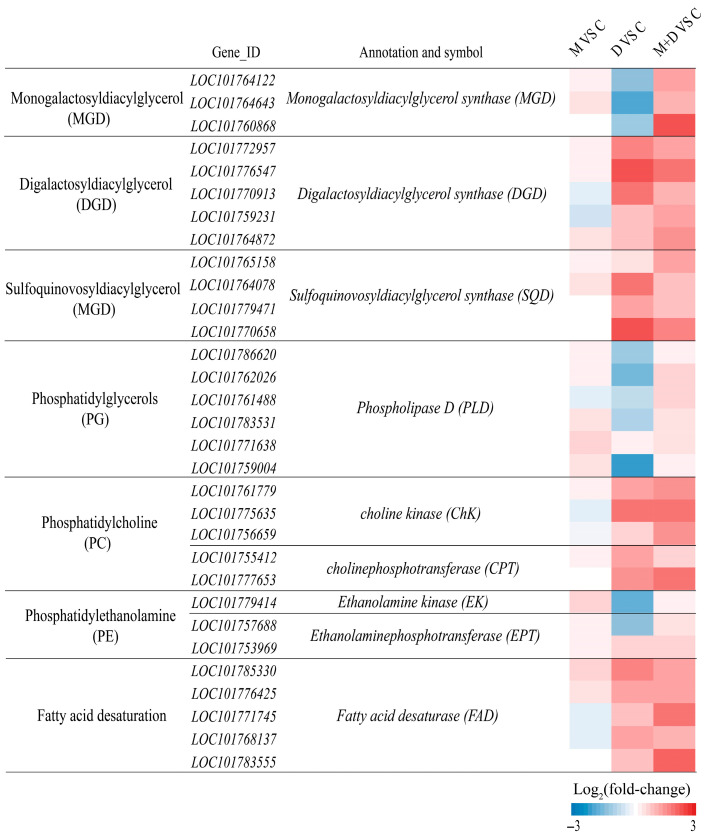
Effects of drought and melatonin on the relative expression levels of lipid biosynthesis-related genes in leaves of foxtail millet. Color scale indicates log_2_(fold-change) values.

In alignment with alterations in membrane lipid content, drought stress markedly suppressed the expression of genes associated with MGDG synthesis, such as *monogalactosyldiacylglycerol synthase (MGD)*, within the glycolipid synthesis pathway (Figure 8). Conversely, the transcription levels of genes involved in DGDG and SQDG synthesis, including *digalactosyldiacylglycerol synthase (DGD)* and *sulfoquinovosyldiacylglycerol synthase (SQD)*, were upregulated. Treatment with exogenous melatonin significantly enhanced the expression of *MGD*, *DGD*, and *SQD* genes (Figure 8). Regarding phospholipid synthesis, drought stress resulted in a reduction in the transcription levels of PG synthesis genes, such as *phospholipase D (PLD)*, and PE synthesis-related genes, including *ethanolamine kinase (EK)* and *ethanolaminephosphotransferase (EPT)*. In contrast, the expression of PC synthesis genes, such as *choline kinase (CHK)* and *cholinephosphotransferase (CPT)*, was significantly elevated. Following melatonin application, the expression of all these phospholipid synthesis genes was further significantly increased (Figure 8). Furthermore, drought stress substantially enhanced the expression of fatty acid desaturase (FAD) gene family members, and exogenous melatonin treatment further augmented the transcription levels of these genes.

## 3. Discussion

Numerous studies have demonstrated that exogenous melatonin can significantly mitigate the growth inhibition of various crops induced by drought stress. In this study, it was observed that melatonin treatment effectively alleviated the inhibitory effects of drought stress on foxtail millet growth, consistent with findings in soybean [12], maize [28], and wheat [29]. The mechanism of action of melatonin can be summarized as follows: its protective role involves preserving photosynthetic organ structure, maintaining ionic homeostasis, upregulating antioxidant enzymes (SOD, POD, CAT), modulating the ascorbate–glutathione cycle, and stabilizing hormonal signaling networks [12,13]. Notably, membrane lipid remodeling, a crucial strategy for plant adaptation to drought stress, was analyzed in this study to elucidate the regulatory role of membrane lipid metabolism in melatonin-mediated drought tolerance.

Photosynthesis in plants is intricately linked to their growth and development. Drought stress adversely affects the processes of photosynthetic carbon assimilation and respiration by disrupting metabolic equilibrium [30]. Under drought stress, photosynthetic parameters, including the net photosynthetic rate and stomatal conductance in maize, exhibit a marked decline [28]. Our research findings confirm that drought stress significantly inhibits the photosynthetic activity in millet leaves (Figure 3A). Notably, the application of melatonin ameliorated the reduction in photosynthetic parameters induced by drought stress. These results align with previous studies conducted on wheat [29] and maize [31]. Chlorophyll content and chlorophyll fluorescence parameters serve as critical indicators for assessing the absorption and conversion of light energy by leaves under adverse stress conditions [32]. Prior study has demonstrated that drought stress can cause damage to chloroplast structure and a decrease in chlorophyll content [32]. In this study, our findings demonstrate that exposure to drought stress results in a marked reduction in chlorophyll content and Fv/Fm (Figure 3D,I). This condition impairs the plant’s capacity to absorb and transfer light energy, thereby inhibiting the utilization of electrons in the photosynthetic electron transport chain for ATP and NADPH synthesis and leading to the production of substantial amounts of reactive oxygen species (ROS) [33]. Previous research has indicated that melatonin can preserve the structural integrity of chloroplasts, enhance photosynthetic efficiency, and effectively mitigate ROS accumulation in tomato leaves subjected to drought stress [34]. Similarly, Ren et al. [35] reported that melatonin significantly enhanced the photosynthetic properties of maize leaves under abiotic stress, primarily by upregulating the expression of genes associated with electron transfer and reducing ROS levels. Our research suggests that the application of melatonin significantly increases the chlorophyll content and Fv/Fm values in foxtail millet leaves under drought stress (Figure 3D,I). These results indicate that melatonin plays a key role in optimizing light energy absorption and conversion in foxtail millet leaves under drought stress.

The cell membrane functions as a critical physical barrier against drought stress, with its structural integrity and functional fluidity being essential for plant adaptability [36]. Drought stress triggers an excessive accumulation of ROS, including O_2_^−^· and H_2_O_2_, within the cells. These ROS subsequently attack the biological macromolecules of the membrane system, such as proteins and lipids, leading to membrane lipid peroxidation and the degradation of macromolecules [14]. This cascade of events results in an abnormal increase in membrane permeability, a loss of structural integrity, and ultimately manifests as ion efflux and an elevated EL. MDA, a characteristic end-product of membrane lipid peroxidation, is frequently utilized in conjunction with EL values to evaluate the drought tolerance of plants [37]. The present study observed a significant increase in the levels of ROS, EL values, and MDA in the leaves of foxtail millet seedlings subjected to drought stress, aligning with trends previously reported in wheat [38] and soybean [12]. Notably, the application of melatonin resulted in a significant reduction in ROS levels, EL values, and MDA contents (Figure 4). This finding aligns with prior research, which has demonstrated that melatonin effectively inhibits ROS accumulation in drought-stressed maize and mitigates oxidative damage [39]. These results suggest that melatonin effectively reduces ROS accumulation and alleviates cell membrane damage induced by drought stress.

Lipid metabolism is integral to plant drought tolerance. Nevertheless, drought stress adversely affects lipid synthesis, evidenced by a reduction in total lipid and phospholipid content across various plant species, including sorghum [21] and wheat [40]. Phospholipids serve as fundamental structural constituents of biological membranes, including plasma membranes, with PC and PE being the predominant phospholipids in plant membrane lipids [41]. This study demonstrated that drought stress significantly enhanced the PC content in leaves while reducing the PE content, aligning with the observed decrease in PE within the plasma membrane under drought stress in white clover [42]. As the principal component of the lipid bilayer, a higher PC:PE ratio facilitates the maintenance of bilayer phase stability and membrane fluidity [43]. This ratio is positively associated with the stress resistance of plants, such as the drought resistance of grapes [44] and the heat tolerance of wheat [45]. Notably, treatment with exogenous melatonin enhanced the synthesis of PC by 26.5% under drought stress, and increased the PC/PE ratio to 25.9% (Figure 5E,I). This finding suggests that melatonin can help preserve the homeostasis of plant leaf membranes under drought stress by modulating phospholipid metabolism.

Drought stress frequently results in a reduction in MGDG levels in plant leaves, which subsequently disrupts the structural and functional integrity of the photosynthetic membrane system [22]. This study revealed a significant decline in MGDG content in the leaves of foxtail millet seedlings subjected to drought stress, whereas the levels of DGDG and SQDG exhibited a marked increase (Figure 5A–C). These findings suggest that DGDG and SQDG are integral to maintaining the stability of chloroplast and thylakoid membranes under conditions of drought stress. In *Arabidopsis thaliana*, the dgd1 mutant, characterized by impaired DGDG synthesis, exhibits hindered assembly of the light-harvesting complex II (LHCII) within the thylakoid membrane, diminished stability of photosystem I (PSI), and a reduced chlorophyll fluorescence lifetime [46]. The enhancement of SQDG content plays a pivotal role in plant salt tolerance. This mechanism extends beyond the structural support of the photosynthetic system, potentially engaging in Ca^2+^-dependent signal transduction pathways. Seigneurin-Berny et al. [47] demonstrated that SQDG can bind to annexin in a Ca^2+^-dependent manner, with this protein family being involved in stress responses by modulating membrane organization, membrane fusion events, and transmembrane ion transport. The study further revealed that exogenous melatonin treatment can increase the biosynthesis of MGDG, DGDG, and SQDG under drought stress (Figure 5A–C). This suggests that melatonin preserves photosynthetic membrane integrity by regulating galactolipid metabolism, thereby enhancing the photosynthetic performance of leaves under stress conditions.

The unsaturation index of fatty acids serves as a crucial indicator of plasma membrane characteristics and is intricately linked to plant adaptability to abiotic stress [48]. In this study, the DBI of DGDG, SQDG, and PC exhibited a significant increase under drought stress, suggesting enhanced membrane fluidity in leaf bilayers (Figure 7). Following exposure to cold conditions, the DBI of all glycerophospholipids was elevated [18]. In leguminous species, treatment with NaCl has been shown to enhance the unsaturation levels of MGDG, DGDG, and PG, potentially facilitating adaptation to salt stress and preserving chloroplast membrane integrity [49]. In the present study, melatonin treatment further increased the unsaturation index of these fatty acids, indicating that melatonin enhances membrane fluidity under drought stress conditions (Figure 7). The degree of unsaturation in fatty acids is regulated by the activity of fatty acid desaturase enzymes (FAD), which introduce double bonds at specific positions along the acyl chain. Within the endoplasmic reticulum, *FAD2* catalyzes the conversion of oleic acid (18:1) to linoleic acid (18:2), and subsequently, *FAD3* further desaturates linoleic acid to produce linolenic acid (18:3). In the plastid, *FAD7* and *FAD8* facilitate the conversion of 18:2 to 18:3 [50]. In transgenic tobacco plants, the overexpression of the ω-3 desaturase enzyme (*FAD3*) has been associated with enhanced tolerance to salt and drought stress [51]. Conversely, knockout of the *FAD8* gene results in reduced membrane fluidity under low-temperature stress conditions [52]. In this study, we observed that the expression of the *FAD* gene was upregulated in response to drought stress, and this upregulation was further enhanced by melatonin treatment (Figure 8). This suggests that melatonin may augment the unsaturation of membrane lipids, thereby increasing membrane fluidity.

## 4. Materials and Methods

### 4.1. Plant Growth Conditions and Drought Treatments

The drought-sensitive foxtail millet variety, *Setaria italica* L. cv. JG21, was used as the experimental material. The seeds underwent surface sterilization using a 1% sodium hypochlorite solution, followed by three rinses with sterile water. The seeds were sown in plastic pots with dimensions of 20 cm in diameter and 19 cm in height. Each pot was filled with 8 kg of yellow loam soil. Fertilizer application was determined per kilogram of dry soil, with 0.12 g of N, 0.1 g of P_2_O_5_, and 0.1 g of K_2_O being administered, respectively. Upon the full expansion of the fifth leaf of the foxtail millet seedlings, a foliar spray treatment was implemented, involving the application of varying concentrations of melatonin (0, 10, 25, 50, 100 μM) or distilled water as a control. Twelve hours subsequent to the melatonin treatment, a drought treatment was initiated using the weighing and water control method [28]. The relative soil water content of the well-watered group was maintained between 70% and 90%, while the drought treatment group experienced a gradual reduction in soil moisture to approximately 40%. The experiment was designed with ten treatment groups: (1) 0 μM melatonin, (2) 10 μM melatonin, (3) 25 μM melatonin, (4) 50 μM melatonin, (5) 100 μM melatonin, (6) drought, (7) drought + 10 μM melatonin, (8) drought + 25 μM melatonin, (9) drought + 50 μM melatonin, and (10) drought + 100 μM melatonin. Each treatment comprised twenty pots, with each box containing three plants. Foxtail millet seedlings were cultivated within an artificial climate chamber set to a day/night temperature of 28 °C/23 °C, a photoperiod of 12 h of light and 12 h of darkness, a light intensity of 800 μmol·m^−2^·s^−1^, and a relative humidity ranging from 45% to 55%. Twelve days following the application of drought treatment, the most recently fully expanded leaf and root samples from each treatment group were collected. Samples were obtained from the leaves of five plants housed in five separate pots, and these were pooled to form independent duplicate samples. Each treatment was represented by three independent biological replicates. The samples were promptly frozen in liquid nitrogen and subsequently stored at −80 °C in an ultra-low temperature freezer for further analysis.

### 4.2. Measurement of Dry Weight and Leaf Area

Following a 12-day period of drought treatment, samples from both the aboveground and root of the foxtail millet plants were collected. Subsequently, these samples were subjected to drying in an oven set at 80 °C until a constant weight was attained, enabling the determination of their dry weights. The leaf area of the plants was quantified using a leaf area meter (CI-203, CID Bio-Science, Camas, WA, USA).

### 4.3. Determination of Endogenous Melatonin Content

The endogenous melatonin concentration was quantified utilizing high-performance liquid chromatography (HPLC, Agilent 1260, Agilent Technologies, Santa Clara, CA, USA), based on the protocol established by Chen et al. [53]. Specifically, 0.5 g of freeze-dried leaf tissue was pulverized in liquid nitrogen and subsequently extracted with 5 mL of methanol. The resulting suspension underwent centrifugation at 4 °C at 10,000× *g* for 30 min, after which the supernatant was evaporated under a nitrogen stream. The residue was then reconstituted in 0.2 mL of a Na_2_HPO_4_: acetonitrile solution (65:35, *v*/*v*) and filtered through a 0.22 μm micropore filter membrane. An injection volume of 5 μL was employed, and chromatographic separation was conducted at 30 °C using a C18 column. The flow rate was maintained at 0.5 mL/min, and detection was performed at a wavelength of 220 nm.

### 4.4. Determination of Chlorophyll Content, Gas Exchange Parameters, and Chlorophyll Fluorescence Parameters

The quantification of chlorophyll content was conducted following the methodology outlined by Sharma et al. [54]. Fresh leaf samples, weighing 0.2 g, were immersed in 95% ethanol until the tissue was completely decolorized. The resulting extract was subjected to centrifugation at 10,000× *g* for 5 min. Absorbance measurements were taken at wavelengths of 645 nm, 652 nm, and 663 nm using a UV-2600 ultraviolet spectrophotometer (Shimadzu, Kyoto, Japan). Gas exchange parameters were assessed using the Li-6800 portable photosynthesis system (LICOR, Lincoln, NE, USA) under a light intensity of 800 μmol·m^−2^·s^−1^, between 09:00 and 11:00 on sunny days. These parameters included the net photosynthetic rate (P_n_), transpiration rate (E_s_), and stomatal conductance (G_s_). The chlorophyll fluorescence parameters were measured using a PAM-2500 modulated chlorophyll fluorescence meter (Walz, Effeltrich, Germany). Prior to measurement, leaves were dark-adapted for 30 min, and six biological replicates were employed.

### 4.5. ROS and Oxidative Damage Assays

The quantification of O_2_^−^· was performed in accordance with the methodology outlined by Jahan et al. [55]. Initially, 0.2 g of fresh root samples were pulverized in liquid nitrogen, followed by the addition of 2 mL of pre-cooled 50 mM phosphate buffer at pH 7.8. The homogenized mixture was then subjected to centrifugation at 12,000× *g* for 20 min at 4 °C. Subsequently, 0.5 mL of the resulting supernatant was combined with 0.1 mL of 10 mM hydroxylamine hydrochloride and 0.5 mL of the buffer, and the mixture was incubated at 25 °C for 30 min. Following this, 1 mL of 17 mM sulfanilic acid solution and 1 mL of 7 mM naphthylamine solution were sequentially added, and the mixture was incubated for an additional 30 min. The absorbance was measured at 530 nm, and the concentration of O_2_^−^· was determined using a NaNO_2_ standard curve. The assessment of H_2_O_2_ was conducted utilizing the procedure described byMuhammad et al. [39]. The determination of MDA and EL was conducted according to the method of Sabzi-Nojadeh et al. [56]. 0.1 g of fresh leaves were added to 2 mL of 0.1% trichloroacetic acid (TCA), and then homogenized. The mixture was centrifuged at 4 °C, 12,000× *g* for 5 min. 350 μL of the supernatant was taken and mixed with 1.4 mL of 20% TCA solution containing 5% thiobarbituric acid (TBA). The mixture was incubated at 96 °C in a water bath for 30 min. After cooling, the supernatant was taken to measure the absorbance at 532 nm. Determination of EL: The leaf samples were placed in a test tube containing 15 mL of distilled water, and left to soak at room temperature for 24 h. The initial conductivity (EC_1_) was then measured. The sample was subjected to autoclaving at 121 °C for 20 min, and the final conductivity (EC_2_) was measured after cooling. The EL (%) was calculated using the formula EL (%) = (EC_1_/EC_2_) × 100.

### 4.6. Determination of Lipid and Fatty Acid Contents

Lipid extraction and separation were conducted following the methodology described by Zheng et al. [57]. Initially, 0.2 g of fresh leaf sample was combined with an extraction solvent comprising chloroform, methanol, and formic acid in a volumetric ratio of 20:10:1, and vortexed for 5 min. Subsequently, 1 mL of a phosphate-buffer solution, consisting of 0.2 M H_3_PO_4_ and 1 M KCl, was added. After brief vortex mixing, the mixture was subjected to centrifugation at 4000× *g* for 3 min at 4 °C, and the lower chloroform phase was collected. This extraction process was repeated twice, and the chloroform phases from all three extractions were combined and dried under a nitrogen stream. Lipid separation was performed using two-dimensional thin-layer chromatography (2D-TLC). Pre-coated silica gel plates (Silica Gel 60, 10 cm × 10 cm, 0.2 mm thickness; Merck, Darmstadt, Germany) with a pore size of 60 Å and particle size of 5–15 μm were used. The silica gel plate was activated according to the procedure outlined by Wang and Benning [56], and 15 μL of the lipid extract was applied. The prepared silica gel plates were placed in a sealed developing chamber, containing a developing solvent system of acetone, toluene, and water in a volumetric ratio of 91:30:7.5, with a total volume of 30 mL. Subsequently, the plate was placed into a different sealed development chamber, where it was developed for the second dimension using a solvent system consisting of acetone, toluene, acetic acid, and water in a volumetric ratio of 95:10:16:7. Once the solvent front was 1 cm from the top of the plate, the silica gel plate was removed and allowed to dry at room temperature. The lipid bands, once separated, were visualized using the iodine. Lipid identification was conducted based on the specific retention factor (Rf) values of standard compounds. Chloroform solutions of PC, PE, PG, PI, MGDG, DGDG, and SQDG were prepared at a concentration of 10 µg/mL each, sourced from Sigma. These standard substances were subjected to the same separation procedure as described previously, and their Rf values were calculated using the formula Rf = (distance traveled by the center of the spot)/(distance traveled by the solvent front).

The fatty acid content was quantified following the methodology outlined by Wang and Benning [58]. The procedure involved scraping the silica gel containing the target lipid into a glass tube, followed by the addition of 1 mL of a 1 N hydrochloric acid–methanol solution and 100 μL of a pentadecanoic acid internal standard (C15:0, 50 μg·mL^−1^). The tube was then sealed and heated in a water bath at 80 °C for 25 min. Upon cooling, 1 mL of 0.9% NaCl and 1 mL of n-hexane were sequentially added. The mixture was centrifuged at 1000× *g* for 3 min at room temperature, after which 800 μL of the n-hexane phase was transferred, evaporated under a nitrogen stream, and subsequently redissolved in 50 μL of n-hexane. Gas chromatography was performed using a TRACE 1310 system (ThermoFisher, Waltham, MA, USA) equipped with a TG-FAME capillary column (50 m × 0.25 mm × 0.2 μm). The carrier gas, helium, was maintained at a flow rate of 0.5 mL·min^−1^ with a split ratio of 1:30. The injection port and detector temperatures were set at 280 °C. The temperature program commenced with a hold at 80 °C for 1 min, followed by a ramp of 20 °C·min^−1^ to 160 °C with a hold for 1.5 min, then a ramp of 3 °C·min^−1^ to 205 °C with a hold for 4 min, and finally a ramp of 3 °C·min^−1^ to 250 °C with a hold for 2 min. A quantitative analysis was performed employing the internal standard method to determine the lipid and fatty acid contents. Additionally, the DBI was calculated following the methodology outlined by Chen et al. [22].

### 4.7. RNA Extraction and qRT-PCR

Following a 12-day drought stress treatment, the most recently fully expanded leaves of foxtail millet seedlings were collected. These samples were immediately flash-frozen in liquid nitrogen and subsequently stored at −80 °C. The qRT-PCR analysis was conducted following the methodology outlined by Ren et al. [35]. Total RNA extraction was conducted using the TRIzol™ reagent (Invitrogen, Carlsbad, CA, USA) in strict accordance with the manufacturer’s protocol. *Siactin* served as the internal reference gene, and relative expression levels were determined via the 2^−ΔΔCT^ method.

### 4.8. Statistical Analysis

Data were presented as the mean ± standard error (mean ± SE), with a minimum of four independent replicates for each experimental group. Statistical analysis was conducted using one-way analysis of variance (ANOVA) via SPSS Statistics 19.0 (IBM, Armonk, NY, USA). Duncan’s multiple range test was employed to assess intergroup differences, with significance determined at *p* < 0.05. Distinct lowercase letter notations were used to denote significant differences between treatments.

## 5. Conclusions

**Figure 9:**
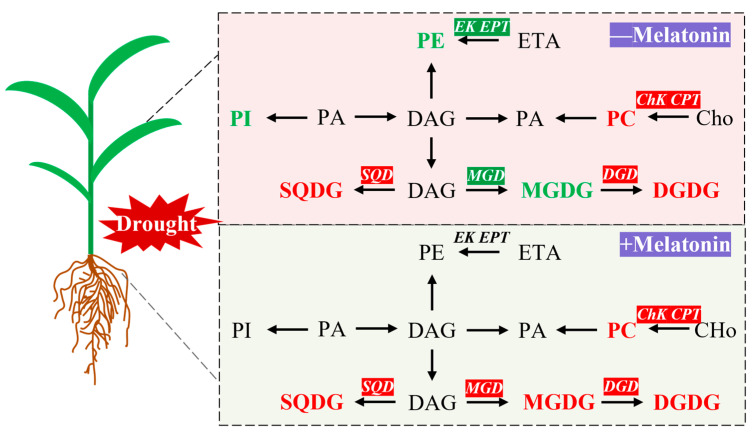
Melatonin improves drought stress tolerance by remodeling lipid metabolism in *Setaria italica* L. Red and green were indicates that genes or lipids were upregulated or downregulated by melatonin, respectively. MGDG: Monogalactosyldiacylglycerol; DGDG: Digalactosyldiacylglycerol; SQDG: Sulfoquinovosyldiacylglycerol; PC: Phosphatidylcholine; PE: Phosphatidylethanolamine; PI: Phosphatidylinositol; DAG: Diacylglycerol; PA: Phosphatidic Acid; ETA: Ethanolamine; Cho: Choline; *MGD*: *Monogalactosyldiacylglycerol synthase*; *DGD*: *Digalactosyldiacylglycerol synthase*; *SQD*: *Sulfoquinovosyldiacylglycerol synthase*; *EK*: *Ethanolamine kinase*; *EPT*: *Ethanolaminephosphotransferase*; *CHk*: *Choline kinase*; *CPT*: *Cholinephosphotransferase*.

Melatonin, recognized as a crucial signaling molecule and antioxidant, has been substantiated by numerous studies to markedly enhance plant drought tolerance. The findings of this investigation demonstrate that, under drought conditions, melatonin treatment effectively augments the photosynthetic attributes of foxtail millet seedlings while mitigating oxidative damage. From the perspective of lipid metabolism, melatonin markedly enhanced the expression levels of the *MGD*, *DGD*, *SQD*, *ChK*, and *CPT* genes. This upregulation led to increased contents of MGDG, DGDG, SQDG, and PC in the leaves, primarily increasing the content of their unsaturated fatty acids, specifically 18:3. These alterations were corroborated by changes observed in the DBI (Figure 9). These findings demonstrate that melatonin enhances membrane stability and fluidity by reprogramming lipid metabolism, thereby effectively improving the drought resistance of foxtail millet seedlings. In conclusion, this study elucidates the mechanism of melatonin-induced drought resistance from the perspective of lipid remodeling, providing a theoretical basis and potential strategies for improving drought resistance in crops and supporting sustainable agriculture in arid regions. Future research should prioritize two directions. First, the high production cost of melatonin remains a significant barrier to its widespread application in agriculture. Currently, melatonin is predominantly produced through animal extraction or chemical synthesis, both of which are associated with high costs and low yields. Recent advancements in microbial synthesis methods have shown promise as a more efficient, safe, and potentially cost-effective alternative for melatonin production. Second, existing studies on the role of melatonin in enhancing crop stress resistance are largely confined to the seedling stage and controlled indoor environments. Future research should focus on validating these findings under field conditions, particularly during critical growth phases such as flowering and grain filling. This would provide a more robust theoretical foundation for the broader agricultural application of melatonin.

## Figures and Tables

**Table 1:** Effects of drought and melatonin on fatty acid composition of lipids in leaves of foxtail millet.

Lipid Classes	Treatments	Fatty Acid Content (nmol mg^−1^ DW)					
		16:0	16:1	18:0	18:1	18:2	18:3
MGDG	C	4.17 ± 0.37 a	0.36 ± 0.03 a	1.94 ± 0.14 a	1.96 ± 0.17 a	1.23 ± 0.06 a	38.31 ± 1.69 a
	M	4.29 ± 0.32 a	0.37 ± 0.03 a	2.06 ± 0.17 a	2.08 ± 0.20 a	1.36 ± 0.13 a	37.65 ± 1.85 a
	D	4.06 ± 0.29 a	0.37 ± 0.02 a	2.04 ± 0.15 a	2.03 ± 0.17 a	1.41 ± 0.11 a	32.18 ± 0.85 c
	DM	4.25 ± 0.33 a	0.41 ± 0.04 a	2.03 ± 0.18 a	2.54 ± 0.24 a	1.39 ± 0.15 a	34.94 ± 1.37 b
DGDG	C	4.68 ± 0.42 a	0.03 ± 0.00 a	2.56 ± 0.24 a	0.46 ± 0.04 a	0.63 ± 0.05 a	16.27 ± 0.68 c
	M	4.53 ± 0.39 a	0.03 ± 0.00 a	2.68 ± 0.27 a	0.48 ± 0.03 a	0.67 ± 0.04 a	15.98 ± 0.92 c
	D	4.72 ± 0.41 a	0.03 ± 0.00 a	2.37 ± 0.21 a	0.51 ± 0.06 a	0.71 ± 0.03 a	19.24 ± 0.74 b
	DM	4.89 ± 0.45 a	0.04 ± 0.00 a	2.67 ± 0.19 a	0.54 ± 0.04 a	0.61 ± 0.07 a	21.37 ± 1.03 a
SQDG	C	3.14 ± 0.15 a	0.12 ± 0.01 a	1.57 ± 0.12 a	0.50 ± 0.05 a	0.49 ± 0.04 a	3.07 ± 0.31 c
	M	3.26 ± 0.20 a	0.13 ± 0.01 a	1.68 ± 0.13 a	0.52 ± 0.03 a	0.54 ± 0.05 a	3.15 ± 0.25 c
	D	3.26 ± 0.17 a	0.14 ± 0.02 a	1.61 ± 0.15 a	0.52 ± 0.04 a	0.58 ± 0.04 a	3.89 ± 0.28 b
	DM	3.08 ± 0.21 a	0.13 ± 0.01 a	1.71 ± 0.13 a	0.49 ± 0.04 a	0.59 ± 0.05 a	4.82 ± 0.26 a
PG	C	1.18 ± 0.05 a	0.56 ± 0.05 a	0.51 ± 0.04 a	0.10 ± 0.01 a	0.25 ± 0.01 a	1.72 ± 0.09 a
	M	1.21 ± 0.09 a	0.57 ± 0.04 a	0.52 ± 0.04 a	0.10 ± 0.01 a	0.28 ± 0.03 a	1.74 ± 0.10 a
	D	1.05 ± 0.05 b	0.46 ± 0.07 a	0.57 ± 0.09 a	0.10 ± 0.01 a	0.27 ± 0.02 a	1.43 ± 0.07 b
	DM	1.28 ± 0.07 a	0.54 ± 0.04 a	0.49 ± 0.04 a	0.10± 0.01 a	0.27 ± 0.02 a	1.44 ± 0.08 b
PC	C	0.83 ± 0.07 a	0.23 ± 0.01 a	0.08 ± 0.01 a	0.09 ± 0.01 a	0.95 ± 0.07 a	1.34 ± 0.11 c
	M	0.84 ± 0.06 a	0.24 ± 0.02 a	0.08 ± 0.01 a	0.10 ± 0.01 a	0.91 ± 0.07 a	1.39 ± 0.07 c
	D	0.89 ± 0.07 a	0.21 ± 0.02 a	0.08 ± 0.00 a	0.08 ± 0.01 a	0.97 ± 0.08 a	1.67 ± 0.10 b
	DM	0.80 ± 0.05 a	0.25 ± 0.01 a	0.09 ± 0.01 a	0.09 ± 0.01 a	0.91 ± 0.09 a	2.03 ± 0.12 a
PE	C	0.31 ± 0.02 a	0.01 ± 0.00 a	0.07 ± 0.01 a	0.04 ± 0.00 a	0.27 ± 0.02 a	0.42 ± 0.03 a
	M	0.29 ± 0.04 a	0.01 ± 0.00 a	0.08 ± 0.01 a	0.04 ± 0.01 a	0.29 ± 0.02 a	0.46 ± 0.03 a
	D	0.21 ± 0.02 b	0.01 ± 0.00 a	0.08 ± 0.01 a	0.04 ± 0.00 a	0.22 ± 0.01 b	0.43 ± 0.04 a
	DM	0.21 ± 0.02 b	0.01 ± 0.00 a	0.08 ± 0.01 a	0.04 ± 0.01 a	0.22 ± 0.02 b	0.41 ± 0.04 a
PI	C	0.54 ± 0.03 a	0.02 ± 0.00 a	0.11 ± 0.01 a	0.10 ± 0.01 a	0.53 ± 0.03 a	0.77 ± 0.04 a
	M	0.57 ± 0.05 a	0.02 ± 0.00 a	0.12 ± 0.01 a	0.11 ± 0.05 a	0.57 ± 0.05 a	0.81 ± 0.06 a
	D	0.42 ± 0.04 b	0.02 ± 0.00 a	0.13 ± 0.04 a	0.11 ± 0.01 a	0.55 ± 0.07 a	0.65 ± 0.05 b
	DM	0.44 ± 0.55 b	0.02 ± 0.00 a	0.13 ± 0.04 a	0.11 ± 0.02 a	0.54 ± 0.04 a	0.67 ± 0.04 b

Different letters denote significant differences (*n* = 3, *p* < 0.05). C: Control; M: Melatonin; D: Drought; DM: Drought + Melatonin.

## Data Availability

The data are contained within the manuscript.

## Abbreviations

The following abbreviations are used in this manuscript:

MGDG	Monogalactosyldiacylglycerol
DGDG	Digalactosyldiacylglycerol
SQDG	Sulfoquinovosyldiacylglycerol
PG	Phosphatidylglycerol
PC	Phosphatidylcholine
PE	phosphatidylethanolamine
PI	Phosphatidylinositol
O_2_^−^·	Superoxide anion
H_2_O_2_	Hydrogen peroxide
MDA	Malondialdehyde
EL	Electrolyte leakage rate
ROS	Reactive Oxygen Species

## References

[1] Zahedi S.M., Karimi M., Venditti A. (2021). Plants adapted to arid areas: Specialized metabolites. Nat. Prod. Res..

[2] Heinemann B., Hildebrandt T.M. (2021). The role of amino acid metabolism in signaling and metabolic adaptation to stress-induced energy deficiency in plants. J. Exp. Bot..

[3] Salvi P., Manna M., Kaur H., Thakur T., Gandass N., Bhatt D., Muthamilarasan M. (2021). Phytohormone signaling and crosstalk in regulating drought stress response in plants. Plant Cell Rep..

[4] Arnao M.B., Hernández–Ruiz J. (2019). Melatonin: A new plant hormone and/or a plant master regulator?. Trends Plant Sci..

[5] Antoniou C., Chatzimichail G., Xenofontos R., Pavlou J.J., Panagiotou E., Christou A., Fotopoulos V. (2017). Melatonin systemically ameliorates drought stress-induced damage in Medicago sativa plants by modulating nitro–oxidative homeostasis and proline metabolism. J. Pineal Res..

[6] Menhas S., Lin D., Zhu S., Hayat S., Aftab T., Liu W., Hayat K. (2025). Melatonin as a multifaceted stress protector in rice: Mechanisms, synergies, and knowledge gaps. J. Plant Physiol..

[7] Colombage R., Singh M.B., Bhalla P.L. (2023). Melatonin and abiotic stress tolerance in crop plants. Int. J. Mol. Sci..

[8] Gao Y., Chen H., Chen D., Hao G. (2023). Genetic and evolutionary dissection of melatonin response signaling facilitates the regulation of plant growth and stress responses. J. Pineal Res..

[9] Ahammed G.J., Li Z., Chen J., Dong Y., Qu K., Guo T., Wang F., Liu A., Chen S., Li X. (2024). Reactive oxygen species signaling in melatonin-mediated plant stress response. Plant Physiol. Biochem..

[10] Dzinyela R., Manda T., Hwarari D., Ramakrishnan M., Ahmad Z., Agassin R.H., Yang L.M., Movahedi A. (2025). Melatonin-mediated phytohormonal crosstalk improves salt stress tolerance in plants. Planta.

[11] Hao X., Ren J., Xu M., Sun B., Li R., Yang S., Xu W. (2025). Melatonin in plant pathogen defense: A review of its role in horticultural crops. Hortic. Res..

[12] Liu X., Chen A., Wei Q., Wang C., Zhao Q., Wang Q., Zheng X., He T., Qi J., Yin Y. (2025). Exogenous melatonin inhibits the expression of *GmABI5* and enhances drought resistance in fodder soybean through an ABA-independent pathway. Plant Cell Environ..

[13] Mao C., Wang L., Mao Y., Li Y., Peng Y., Fan Y., Li J., Zhu Y., Xu X., Li P. (2025). Melatonin integrates multiple biological and phytohormonal pathways to enhance drought tolerance in rice. Planta.

[14] Li X., Zhao Y., Gao C., Li X., Wu K., Lin M., Sun W. (2025). Integrated analysis of physiological responses and transcriptome of cotton seedlings under drought stress. Int. J. Mol. Sci..

[15] Yu L., Zhou C., Fan J., Shanklin J., Xu C. (2021). Mechanisms and functions of membrane lipid remodeling in plants. Plant J..

[16] Sharma P., Lakra N., Goyal A., Ahlawat Y.K., Zaid A., Siddique K.H.M. (2023). Drought and heat stress mediated activation of lipid signaling in plants: A critical review. Front. Plant Sci..

[17] Xu X., Zhang J., Yan B., Wei Y., Ge S., Li J., Han Y., Li Z., Zhao C., Xu J. (2021). The adjustment of membrane lipid metabolism pathways in maize roots under saline-alkaline stress. Front. Plant Sci..

[18] Zheng Y., Xia Z., Wu J., Ma H. (2021). Effects of repeated drought stress on the physiological characteristics and lipid metabolism of *Bombax ceiba* L. during subsequent drought and heat stresses. BMC Plant Biol..

[19] Olmedo P., Núñez-Lillo G., Toro G., Opazo I., Salvatierra A., Meneses C., Pedreschi R., Pimentel P. (2025). Integration of lipidomics and transcriptomics provides new insights into lipid metabolism in response to water deficit in *Prunus* spp. rootstock leaves. Environ. Exp. Bot..

[20] Okazaki Y., Saito K. (2014). Roles of lipids as signaling molecules and mitigators during stress response in plants. Plant J..

[21] Xu D., Ni Y., Zhang X., Guo Y. (2022). Multiomic analyses of two sorghum cultivars reveals the change of membrane lipids in their responses to water deficit. Plant Physiol. Biochem..

[22] Chen D., Wang S., Qi L., Yin L., Deng X. (2018). Galactolipid remodeling is involved in drought-induced leaf senescence in maize. Environ. Exp. Bot..

[23] Zhang D., Li J., Li M., Cheng Z., Xu Q., Song X., Shang X., Guo W. (2022). Overexpression of a cotton nonspecific lipid transfer protein gene, *GhLTP4*, enhances drought tolerance by remodeling lipid profiles, regulating abscisic acid homeostasis and improving tricarboxylic acid cycle in cotton. Environ. Exp. Bot..

[24] Yang Z., Zhang H., Li X., Shen H., Gao J., Hou S., Zhang B., Mayes S., Bennett M., Ma J. (2020). A mini foxtail millet with an *Arabidopsis*-like life cycle as a C4 model system. Nat. Plants.

[25] Han Y., Yi H. (2025). Sulfate enhances drought tolerance in foxtail millet seedlings by promoting ABA biosynthesis and inducing stomatal closure. J. Plant Growth Regul..

[26] Farooq M., Gogoi N., Barthakur S., Baroowa B., Bharadwaj N., Alghamdi S.S., Siddique K.H.M. (2017). Drought stress in grain legumes during reproduction and grain filling. J. Agron. Crop Sci..

[27] Xuan H., Huang Y., Zhou L., Deng S., Wang C., Xu J., Wang H., Zhao J., Guo N., Xing H. (2022). Key soybean seedlings drought-responsive genes and pathways revealed by comparative transcriptome analyses of two cultivars. Int. J. Mol. Sci..

[28] Ren J., Yang X., Ma C., Wang Y., Zhao J. (2021). Melatonin enhances drought stress tolerance in maize through coordinated regulation of carbon and nitrogen assimilation. Plant Physiol. Biochem..

[29] Zhang Y., Li Y., Liu J., Suo L., Li D., He L., Duan J., Wang Y., Feng W., Guo T. (2025). Exogenous melatonin alleviates drought stress in wheat by enhancing photosynthesis and carbon metabolism to promote floret development and grain yield. Plant Stress.

[30] He J., Hu W., Li Y., Zhu H., Zou J., Wang Y., Meng Y., Chen B., Zhao W., Wang S. (2022). Prolonged drought affects the interaction of carbon and nitrogen metabolism in root and shoot of cotton. Environ. Exp. Bot..

[31] Wang Y., Zhou W., Wang Z., Gao S., Zhang R. (2025). Integrated metabolome, transcriptome and physiological analyses of melatonin-induced drought responses in maize roots and leaves. Plant Growth Regul..

[32] Zahra N., Hafeez M.B., Kausar A., Al Zeidi M., Asekova S., Siddique K.H., Farooq M. (2023). Plant photosynthetic responses under drought stress: Effects and management. J. Agron. Crop Sci..

[33] Zhang Z., Cao B., Gao S., Xu K. (2019). Grafting improves tomato drought tolerance through enhancing photosynthetic capacity and reducing ROS accumulation. Protoplasma.

[34] Altaf M.A., Shahid R., Ren M.X., Naz S., Altaf M.M., Khan L.U., Tiwari R.K., Lal M.K., Shahid M.A., Kumar R. (2022). Melatonin improves drought stress tolerance of tomato by modulating plant growth, root architecture, photosynthesis, and antioxidant defense system. Antioxidants.

[35] Ren J., Yang X., Zhang N., Feng L., Ma C., Wang Y., Yang Z., Zhao J. (2022). Melatonin alleviates aluminum-induced growth inhibition by modulating carbon and nitrogen metabolism, and reestablishing redox homeostasis in *Zea mays* L.. J. Hazard. Mater..

[36] Li Y.K., Zhang Y.M., Dai G.Y., Chen Y.L., Chen D.K., Yao N. (2025). Sphingolipid remodeling in the plasma membrane is essential for osmotic stress tolerance in *Arabidopsis*. Plant Physiol..

[37] Akter N., Brishty T.A., Karim M.A., Ahmed M.J.U., Islam M.R. (2023). 2023. Leaf water status and biochemical adjustments as a mechanism of drought tolerance in two contrasting wheat (*Triticum aestivum* L.) varieties. Acta Physiol. Plant..

[38] Wang J., Zhang X., Han Z., Feng H., Wang Y., Kang J., Han X., Wang L., Wang C., Li H. (2022). Analysis of physiological indicators associated with drought tolerance in wheat under drought and re-watering conditions. Antioxidants.

[39] Muhammad I., Yang L., Ahmad S., Farooq S., Khan A., Muhammad N., Ullah S., Adnan M., Ali S., Liang Q.P. (2023). Melatonin-priming enhances maize seedling drought tolerance by regulating the antioxidant defense system. Plant Physiol..

[40] Wang Y., Zhang X., Huang G., Feng F., Liu X., Guo R., Gu F., Zhong X., Mei X. (2020). Dynamic changes in membrane lipid composition of leaves of winter wheat seedlings in response to PEG-induced water stress. BMC Plant Biol..

[41] Larsson K.E., Nyström B., Liljenberg C. (2006). A phosphatidylserine decarboxylase activity in root cells of oat (*Avena sativa*) is involved in altering membrane phospholipid composition during drought stress acclimation. Plant Physiol. Biochem..

[42] Hassan M.J., Qi H., Cheng B., Hussain S., Peng Y., Liu W., Feng G.Y., Zhao J.M., Li Z. (2022). Enhanced adaptability to limited water supply regulated by diethyl aminoethyl hexanoate (DA-6) associated with lipidomic reprogramming in two white clover genotypes. Front. Plant Sci..

[43] Gao Y., Li M., Zhang X., Yang Q., Huang B. (2020). Up-regulation of lipid metabolism and glycine betaine synthesis are associated with choline-induced salt tolerance in halophytic seashore paspalum. Plant Cell Environ..

[44] Toumi I., Gargouri M., Nouairi I., Moschou P.N., Salem-Fnayou A.B., Mliki A., Zarrouk M., Ghorbel A. (2008). Water stress induced changes in the leaf lipid composition of four grapevine genotypes with different drought tolerance. Biol. Plant..

[45] Narayanan S., Tamura P.J., Roth M.R., Prasad P.V., Welti R. (2016). Wheat leaf lipids during heat stress: I. High day and night temperatures result in major lipid alterations. Plant Cell Environ..

[46] Klaus D., Hartel H., Fitzpatrick L.M., Froehlich J.E., Hubert J., Benning C., Dormann P. (2002). Digalactosyldiacylglycerol synthesis in chloroplasts of the Arabidopsis *dgd1* mutant. Plant Physiol..

[47] Seigneurin-Berny D., Rolland N., Dorne A.J., Joyard J. (2000). Sulfolipid is a potential candidate for annexin binding to the outer surface of chloroplast. Biochem. Biophys. Res. Commun..

[48] Liu X., Ma D., Zhang Z., Wang S., Du S., Deng X., Yin L. (2019). Plant lipid remodeling in response to abiotic stresses. Environ. Exp. Bot..

[49] Hori K., Nobusawa T., Watanabe T., Madoka Y., Suzuki H., Shibata D., Shimojima M., Ohta H. (2016). Tangled evolutionary processes with commonality and diversity in plastidial glycolipid synthesis in photosynthetic organisms. BBA-Mol. Cell Biol. L..

[50] Ge S., Liu D., Chu M., Liu X., Wei Y., Che X., Zhu L., He L., Xu J. (2022). Dynamic and adaptive membrane lipid remodeling in leaves of sorghum under salt stress. Crop J..

[51] Zhang M., Barg R., Yin M., Gueta-Dahan Y., Leikin-Frenkel A., Salts Y., Shabtai S., Ben-Hayyim G. (2005). Modulated fatty acid desaturation via overexpression of two distinct ω-3 desaturases differentially alters tolerance to various abiotic stresses in transgenic tobacco cells and plants. Plant J..

[52] Tovuu A., Zulfugarov I.S., Wu G., Kang I.S., Kim C., Moon B.Y., An G., Lee C.H. (2016). Rice mutants deficient in ω-3 fatty acid desaturase (FAD8) fail to acclimate to cold temperatures. Plant Physiol. Biochem..

[53] Chen G., Huo Y., Tan D.X., Liang Z., Zhang W., Zhang Y. (2003). Melatonin in Chinese medicinal herbs. Life Sci..

[54] Sharma A., Wang J., Xu D., Tao S., Chong S., Yan D., Li Z., Yuan H., Zheng B., Zheng B. (2020). Melatonin regulates the functional components of photosynthesis, antioxidant system, gene expression, and metabolic pathways to induce drought resistance in grafted *Carya cathayensis* plants. Sci. Total Environ..

[55] Jahan M.S., Guo S., Baloch A.R., Sun J., Shu S., Wang Y., Ahammed G.J., Kabir K., Roy R. (2020). Melatonin alleviates nickel phytotoxicity by improving photosynthesis, secondary metabolism and oxidative stress tolerance in tomato seedlings. Ecotoxicol. Environ. Saf..

[56] Sabzi-Nojadeh M., Pouresmaeil M., Amani M., Younessi-Hamzekhanlu M., Maggi F. (2024). Colonization of *Satureja hortensis* L. (*Summer savory*) with *Trichoderma harzianum* alleviates salinity stress via improving physio-biochemical traits and biosynthesis of secondary metabolites. Ind. Crop. Prod..

[57] Zheng Q., Hu J., Dong C., Hu H., Zhao C., Lei K., Tian Z., Dai T. (2024). Differences in membrane lipid homeostasis confer contrast tolerance to low phosphorus in two wheat (*Triticum aestivum* L.) cultivars. Environ. Exp. Bot..

[58] Wang Z., Benning C. (2011). Arabidopsis thaliana polar glycerolipid profiling by thin layer chromatography (TLC) coupled with gas-liquid chromatography (GLC). J. Vis. Exp..

